# Advances in Elucidating the Mitochondrial DNA Mechanisms Underlying Ozone-Induced Inflammation

**DOI:** 10.3390/toxics14030248

**Published:** 2026-03-12

**Authors:** Qianhui Chen, Hao Liu, Junhe Zhou, Yongjie Wei, Lingyan He

**Affiliations:** 1 School of Environment and Energy, Peking University Shenzhen Graduate School, Shenzhen 518055, China; 2501212895@stu.pku.edu.cn; 2 School of Environment, Harbin Institute of Technology, Harbin 150001, China; liuhao222@mails.ucas.ac.cn; 3 State Key Laboratory of Environmental Criteria and Risk Assessment, Chinese Research Academy of Environmental Sciences, Beijing 100012, China; weiyj@craes.org.cn; 4 School of Medicine, Sun Yat-Sen University, Shenzhen 518107, China; zhoujh88@mail2.sysu.edu.cn

**Keywords:** ozone exposure, mtDNA, innate inflammation, mtDNA release

## Abstract

Ground-level ozone is widely acknowledged as one of the primary air pollutants, capable of inducing adverse health effects across multiple human systems, including asthma, cardiovascular events, and central nervous system dysfunction. Epidemiological and toxicological studies indicate that the onset of related systemic diseases is often attributed to ozone-mediated inflammatory responses. However, since O_3_ itself lacks antigenic properties to trigger innate immune responses, an intermediary substance induced by ozone exposure likely activates subsequent inflammatory pathways. Multiple ozone exposure studies have identified mitochondrial DNA (mtDNA) as a potential biomarker released during ozone-induced mitochondrial dysfunction. mtDNA may serve as a damage-related molecular pattern that activates innate immune responses, potentially acting as a crucial link between ozone and inflammatory reactions. This review therefore examines the structure and function of mitochondrial DNA, along with potential mediating mechanisms underlying inflammation associated with ozone exposure.

## 1. Introduction

Air pollution has been proven to cause multiple harms to human health and is recognized as a public health issue [1,2]. The World Health Organization has ranked it as the fourth leading cause of death worldwide [3]. Due to its adverse effects on humans and ecosystems, ground-level ozone (O_3_) has been designated as one of the primary regulated air pollutants by many countries [4]. The primary sources of ground-level O_3_ are linked to human production activities, with nitrogen oxide (NO_x_), volatile organic compounds (VOCs), and carbon monoxide (CO) from transportation emissions and industrial exhaust serving as key precursors. Between 2000 and 2019, concentrations of sulfur dioxide (SO_2_), nitrogen oxides (NO_x_), lead, inhalable particulate matter (PM_10_, aerodynamic diameter ≤ 10 μm), and fine particulate matter (PM_2.5_, aerodynamic diameter ≤ 2.5 μm) showed declining trends globally. However, with industrial and urban development, the level of O_3_ exposure among global urban populations continues to rise at an annual rate of 0.8% [5]. Due to its strong oxidizing properties and high reactivity, O_3_ tends to participate in various environmental reactions and generate reactive oxygen species (ROS) [6]. Consequently, when humans inhale or come into direct contact with O_3_, it reacts with surfactants on mucosal surfaces, causing irritation and damage to the skin and respiratory tract [7]. The damage caused by O_3_ extends beyond the respiratory system. Numerous epidemiological studies indicate that once O_3_ enters the circulatory system, it adversely affects cardiovascular health, increasing the risk of cardiovascular diseases and even leading to premature mortality [8]. Previous research has linked O_3_-induced damage to the respiratory and cardiovascular systems, with O_3_-triggered oxidative stress and peripheral tissue inflammatory responses [9]. Nevertheless, given that O_3_ itself is not an antigen recognized by the immune system to activate the innate immune system, the biological mechanisms by which it induces peripheral tissue inflammation remain a research gap.

Mitochondria, as vital organelles within eukaryotic cells, serve as key units for energy production and metabolism [10], while also playing essential roles in signaling and cellular aging processes [11,12]. Human mitochondria possess unique mitochondrial DNA (mtDNA), a circular double-stranded DNA (dsDNA) consisting of 16, 569 base pairs that encodes 14 polypeptides, 22 tRNAs and 2 rRNAs [13]. Increasing research highlights mtDNA’s importance as an innate immune activator in aging, apoptosis, and inflammatory response activation [14]. Under normal conditions, mtDNA rarely escapes the mitochondria. However, when mitochondria are damaged by reactive oxygen species (ROS) generated during energy production processes or by external environmental stimuli [15,16], mtDNA is released into the cytoplasm. There, it functions as a damage-associated molecular pattern (DAMP) to activate pattern recognition receptors (PRRs), ultimately triggering downstream inflammatory responses [17,18]. This mechanism has been demonstrated in aging and certain metabolic diseases [19]. Additionally, studies have demonstrated that ozone exposure (0.5 ppm) induces cell adhesion and mitochondrial DNA (mtDNA) leakage into the cytoplasm, thereby promoting corneal inflammatory responses. This confirms the pivotal role of mtDNA in ozone-induced inflammatory reactions [20]. This review elucidates the structure and function of mtDNA, summarizes its involvement in specific signaling pathways of innate immune response, and focuses on the mechanisms of mtDNA damage and release triggered by O_3_ exposure or reactive oxygen species (ROS) stimulation. Ultimately, it aims to establish new directions for deepening ozone exposure research and optimizing exposure protocols through systematic analysis.

## 2. The Relationship Between Ozone Exposure and Mitochondrial Function

### 2.1. Health Effects of Ozone

Currently, research on the health effects of O_3_ remains extensive. Substantial evidence indicates that long-term exposure to ozone damages the respiratory, cardiovascular, and nervous systems and correlates with mortality from systemic diseases. Epidemiological reports indicate that O_3_ exposure may also affect human metabolism and fertility, such as inducing diabetes and fetal growth restriction [21,22]. A randomized cross-over exposure study revealed that acute O_3_ exposure can trigger fluctuations across multiple biological pathways, including mitochondrial dysfunction, inflammatory response mechanisms, and neutrophil activation [23]. Animal studies indicate that acute O_3_ exposure correlates with elevated neutrophil counts in mice and altered airway macrophage transcriptomes, predominantly manifested as changes in inflammatory mediator expression levels [24]. From a toxicological perspective, oxidative stress and inflammation are often regarded as the two core mechanisms underlying O_3_-induced injury [25,26].

High concentrations of O_3_ can directly irritate the skin and mucous membranes, causing damaging conditions such as skin allergies, hyperpigmentation, and conjunctivitis [27,28]. Furthermore, studies indicate that O_3_ reacts with oils on the skin surface to produce hydroxyl free radicals, which may further interact with organic compounds, posing potential toxicity to the skin [29]. Similarly, O_3_ can enter the lungs through respiration, leading to an accumulation of reactive oxygen species (ROS) in the respiratory tract and directly increasing the risk of related diseases such as rhinitis, asthma, and chronic obstructive pulmonary disease (COPD) [30]. Certain populations exhibit heightened susceptibility to O_3_ exposure, including infants, the elderly, individuals with pre-existing conditions, and pregnant women [31,32,33]. Studies confirm that short-term acute O_3_ exposure in healthy individuals impairs lung function, disturbs heart rate variability, and activates platelets, leading to elevated blood pressure [34,35]. Long-term exposure to O_3_ further alters the expression of pulmonary inflammatory factors, thereby disrupting both pro-inflammatory and anti-inflammatory regulatory mechanisms and exacerbating health risks [36,37]. Animal studies similarly confirm ozone’s toxic effects. In healthy mice, O_3_ exposure enhances mitochondrial reactive oxygen species (mtROS) production, activates the inflammasome, and induces airway hyper-reactivity and remodeling [38,39,40]. Multi-generational studies in Drosophila reveal that O_3_ exposure adversely affects locomotor activity, body weight, stress resistance, and oxidative stress levels in the third-generation offspring, leading to irreversible metabolic dysfunction [41].

### 2.2. The Effect of Ozone Exposure on Mitochondrial Function

Mitochondria serve as a critical target for ozone-induced cellular damage, with exposure triggering comprehensive structural and functional deterioration. Ozone induces excessive reactive oxygen species production, overwhelming mitochondrial antioxidant capacity and causing lipid peroxidation, membrane potential collapse, and cristae disruption [6,42]. These structural alterations impair respiratory chain complexes and ATP synthesis, compromising cellular energy metabolism [6]. Consequently, damaged mitochondria release cytochrome c and activate caspase-dependent apoptosis, while simultaneously triggering NLRP3 inflammasome-mediated pyroptosis [15]. The PINK1/Parkin-mediated mitophagy pathway is activated to eliminate dysfunctional mitochondria; however, persistent ozone exposure exhausts these compensatory mechanisms [15]. Such mitochondrial damage propagates tissue injury in the lungs, cardiovascular system, and central nervous system, thereby contributing to inflammation, atherogenesis, and neurodegeneration [43].

The prevailing academic view holds that O_3_ exerts its toxic effects through certain reactive molecules generated after permeating the lungs. Historically, lipid peroxidation (LPO) was regarded as a key intermediate in this process. However, recent studies have revealed the crucial role of mitochondria and mitochondrial DNA (mtDNA). In O_3_-induced bronchial inflammation, mitochondrial dysfunction serves as a driving factor for airway remodeling [42]. Furthermore, previous studies indicate that O_3_ exposure correlates with increased copy numbers of circulating mtDNA in peripheral blood [44]. Direct acute O_3_ exposure also induces mtDNA release in mice, activating the cGAS-STING signaling pathway and subsequent inflammatory responses, ultimately leading to conjunctivitis [20]. Multiple studies indicate that mtDNA plays a significant role in O_3_ exposure responses, but the precise mechanisms by which it exerts effects under these conditions require further investigation.

## 3. Structure and Function of mtDNA

### 3.1. Structure and Function of Mitochondria

Mitochondria serve as the primary energy-producing units within cells, generating approximately 95% of cellular energy required for normal physiological activities [45]. Beyond energy production, mitochondria play a pivotal role in regulating numerous other cellular functions, including cell proliferation, apoptosis, and intracellular calcium homeostasis. Mitochondria are dynamic double-membrane organelles whose structural integrity depends on the density of the inner membrane cristae—the region housing respiratory chain complexes and maintaining oxidative phosphorylation capacity [46,47]. Mitochondria are also unique semi-autonomous organelles capable of independent transcription and translation guided by their own genome (mtDNA). Given mtDNA’s central role in these processes, damage or oxidative stress can lead to mitochondrial dysfunction, subsequently affecting a range of cellular functions [48]. Comprehensive quality assessment requires integrated evaluation of morphological structures via electron microscopy, respiratory function through oxygen consumption rates, and membrane potential (ΔΨm) using fluorescent probes [49,50]. Critically, ΔΨm and mitochondrial DNA (mtDNA) exhibit an interdependent relationship: stable ΔΨm is essential for mtDNA replication and nucleoid stability, whereas ΔΨm collapse induces mtDNA oxidation, fragmentation, and subsequent release into the cytoplasm [6,51]. Conversely, damaged mtDNA can exacerbate oxidative stress, further compromising membrane integrity and electrochemical gradients. Therefore, reliable quality evaluation necessitates simultaneous assessment of cristae structure, ΔΨm integrity, and mtDNA stability to accurately determine mitochondrial health and predict downstream inflammatory consequences [6,47].

### 3.2. Structure of mtDNA

The mitochondrial genome (mtDNA) typically adopts a circular, double-stranded DNA structure, comprising 16,569 base pairs with a molecular mass of 107 daltons [52]. mtDNA consists of a guanine-enriched heavy chain (H-chain) and light chain (L-chain) [53]. Due to the absence of introns within its genome, it is often regarded as a more primitive structure relative to the nuclear genome. mtDNA replication initiates at the displacement loop (D-Loop) and is regulated by mitochondrial transcription factor A (TFAM). TFAM typically binds to mtDNA, covering nearly its entire region, and forms a nuclear-like structure localized to the mitochondrial matrix with mtDNA. This ensures the integrity and stability of mtDNA [54,55].

**Figure 1:**
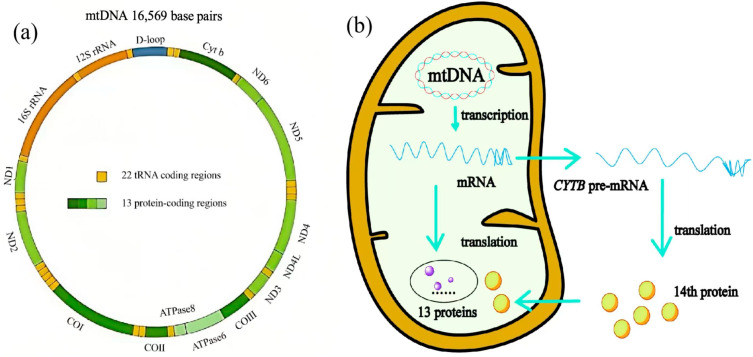
(**a**) Schematic diagram of mitochondrial DNA (mtDNA) structure and function. (**b**) Schematic diagram of the 14th protein expressed from mtDNA. CYTB pre-mRNA can be released into the cytoplasm, where it utilizes the codon template commonly used for translation completion in eukaryotic cells, thereby facilitating the expression of the 14th protein encoded by mtDNA.

As shown in Figure 1, mtDNA can transcribe multiple products that participate in numerous biochemical reactions within mitochondria and the cytoplasm. mtDNA contains 37 genes, including 22 genes encoding tRNAs, 2 genes encoding rRNA, and 13 genes encoding proteins [13,56]. Previous studies generally held that the 13 mRNAs encoded by mtDNA could only be translated by mitochondrial ribosomes. Their standard isoleucine codon differs from cytoplasmic translation, and they serve as subunits of the oxidative phosphorylation (OXPHOS) system [57]. Although most mitochondrial proteins are encoded by the nuclear genome and subsequently transported to mitochondria, a recent discovery revealed that a fourteenth protein encoded by mtDNA is translated in the cytoplasm and plays a crucial role in early development. Furthermore, extensive research has confirmed that mtDNA-derived RNAs can be exported from mitochondria and participate in various physiological and pathological processes [58]. For example, the antisense noncoding mitochondrial RNA (ASncmtRNA) derived from the mitochondrial 16S rRNA gene has been shown to be closely associated with apoptosis and the cell cycle [59]. Research has also revealed that ASncmtRNA knockdown induces cell death in multiple human breast cancer cell lines, preceded by impaired proliferation [60]. Certain mitochondrial-associated noncoding RNAs have also been implicated in cardiac remodeling and coronary artery disease, such as long intergenic noncoding RNA predicting cardiac remodeling (LIPCAR, lncRNA uc022bqs) and long noncoding RNA cytochromeB (LncCYTB) [61,62]. These studies indicate that mitochondrial DNA not only regulates mitochondrial function—and thereby cellular function—by encoding substances required for OXPHOS, transcription, and translation but also directly encodes products involved in cytoplasmic or extracellular matrix reactions.

### 3.3. Functional Characteristics of Intracellular mtDNA

#### 3.3.1. Copy Number and Abundance of mtDNA

The abundance of mtDNA varies across different tissues but remains relatively stable overall [63,64]. The number of mitochondria within a cell depends on its energy demands and the functional role of the cell. For instance, an oocyte may contain hundreds of thousands of mitochondria, while a single white blood cell may harbor fewer than a hundred [65,66]. Mitochondrial numbers also fluctuate within the same cell line, influenced by multiple factors including cell cycle, aging, environment, and redox balance. Furthermore, individual mitochondria may contain multiple copies of mtDNA. Its abundance is not only directly related to mitochondrial transcript levels but also regulated by various proteins encoded by the nuclear genome, such as transcription factors (TFAM and TF2BM) and mitochondrial RNA polymerase (POLRMT) [67,68]. Simultaneously, mtDNA replication is regulated by cellular ATP levels and transcript turnover rates, revealing the critical role of mtDNA methylation in controlling mtDNA abundance [69,70]. Due to the absence of histone protection, mtDNA base pairs remain exposed and vulnerable to environmental factors, explaining the uncertainty and variability in mtDNA copy numbers within individual cells [71,72].

Naturally, mitochondrial DNA abundance or copy number within the same tissue site can serve as a biomarker for mitochondrial dysfunction [73]. Although the copy number of mtDNA varies, the nuclear genome content within the same cell is typically constant. Convenient protocols exist for determining the mitochondrial-to-nuclear genome ratio, denoted as Mt/N to indicate the relative mtDNA content [74]. Mt/N values for specific cell types generally fall within a reasonable range [75] but increase under oxidative stress conditions and show significant differences in various diseases, such as aging, diabetes, cancer, and some neurodegenerative diseases [76,77,78]. However, measuring mtDNA copy numbers in target organs or tissues is relatively challenging in epidemiological studies, leading to the common use of peripheral blood mtDNA content as a biomarker [79]. The application of mtDNA copy numbers as an environmental exposure biomarker has also gained widespread recognition [80]. For instance, studies have demonstrated that ozone exposure damages mitochondria, resulting in increased mtDNA copy numbers in human peripheral blood [44].

#### 3.3.2. mtDNA and Mitochondria Heteroplasmy

Due to the absence of histone protection, the mitochondrial genome (mtDNA) is highly susceptible to mutations [81]. The coexistence of different mtDNA variants within a cell is termed mtDNA heteroplasmy. Given the high copy number of mtDNA, a standard assessment method involves determining the proportion of mtDNA copies carrying a specific mutation relative to the total mtDNA copy number. This metric is referred to as the heterogeneity frequency [82]. Low-level mitochondrial mutations have minimal impact on cellular function, but persistent environmental stressors or aging can lead to the accumulation of such mutations. When the heteroplasmy frequency exceeds a threshold, mitochondrial dysfunction ensues, ultimately resulting in the manifestation of mitochondrial disease phenotypes [83]. Similar to Mt/N ratio, the threshold for mtDNA heterogeneity frequency typically varies between cell types [84].

Mutations leading to increased mtDNA heterogeneity may have multiple underlying causes. First, mtDNA is located adjacent to the mitochondrial membrane, where the vast majority of reactive oxygen species (ROS) are generated alongside energy metabolism [85]. Second, mtDNA replicates through a process known as the chain-displacement model, an asymmetric replication route where the H-strand DNA replicate prior to the L-strand. Compared to the L strand, the H strand remains single-stranded for a longer period, potentially leading to spontaneous deamination of nucleotides within the H-strand [86]. This phenomenon induces mismatches during mtDNA replication, resulting in point mutations that exacerbate internal mtDNA heterogeneity [46]. Furthermore, deoxyribonucleotide triphosphates (dNTPs) exhibit asymmetric distribution within mitochondria. Elevated dGTP levels during mitochondrial DNA replication reduce replication fidelity, thereby increasing the spontaneous mutation rate of mtDNA [87,88,89]. Overall, cellular aerobic respiration and ROS can directly damage mtDNA bases, and this effect is independent of the mtDNA replication pattern, ultimately leading to increased mtDNA heteroplasmy [90].

The abundance of mitochondrial DNA serves as an indicator of tissue metabolic and functional status [91,92]. When tissue cells suffer functional or structural damage, mtDNA escapes into the extracellular environment under stress conditions, thereby triggering systemic adaptation responses to this stress [93]. Recent studies confirm that mtDNA release occurs in various diseases, including neurodegenerative disorders, autoimmune conditions, cardiovascular diseases, and even the aging process. This phenomenon is increasingly recognized as a key biomarker and important trigger for these diseases [94,95,96]. Furthermore, studies using a mouse model of ozone-induced conjunctivitis revealed that mtDNA activation triggers downstream cGAS-STING signaling pathways, ultimately activating innate immune responses [20].

#### 3.3.3. mtDNA and Mitochondrial Dynamics

Mitochondria achieve self-replication and renewal through frequent division and fusion, a process in which morphological changes are termed mitochondrial dynamics. Research confirms that mitochondrial dynamics is closely linked to multiple physiological functions at both the mitochondrial and cellular levels, including cell cycle regulation, apoptosis, mitochondrial autophagy, and embryonic development [97,98,99,100]. Similarly, mitochondrial morphological changes are subject to multifaceted protein regulation via nuclear genome transcription [101]. For instance, mitochondrial fusion proteins (Mnf1 and Mnf2) and optic atrophy-related protein 1 (OPA1) mediate mitochondrial fusion, while dynamin-related protein (Drp1), mitochondrial fission protein 1 (Mfp1), and fission protein 1 (Fis1) mediate mitochondrial fission [101]. The expression of these proteins varies under the influence of multiple factors, including developmental stage and age, cell type, environmental influences, and genetic background. This also indicates that mitochondrial dynamics play a crucial role in adapting to changes in the extracellular environment [102].

Recent evidence indicates that mitochondrial dynamics are closely linked to the maintenance of mtDNA copy numbers, damage repair, and mutation transmission [103,104]. When the heteroplasmy or copy number of mtDNA exceeds normal thresholds, mitochondrial morphology, mass density, and function may alter significantly compared to normal mitochondria. These abnormal mitochondria undergo selective clearance to ensure the stability of the mitochondrial genome. The most well-established pathway is PTEN-induced kinase 1 (PINK1)/Parkin-mediated mitochondrial autophagy, triggered by the accumulation of defective oxidative phosphorylation system components [105]. PINK-1/parkin also participates in mediating the formation of mitochondrial-derived vesicles, thereby promoting the clearance of dysfunctional mitochondria [106]. Furthermore, the inhibition of mitochondrial fission has also been reported to facilitate the physical isolation and removal of mutated or damaged mtDNA, thereby reducing mitochondrial heteroplasmy [107]. Conversely, frequent fission, fusion, and autophagy can disrupt mitochondrial integrity, leading to reactive oxygen species (ROS) leakage or generation within the mitochondrial matrix. This, in turn, compromises telomeres and cell cycle regulators, thereby influencing mitochondrial dynamics through effects on cell cycle regulation [108]. This process also suggests that environmental factors may exert potential impacts on mitochondrial dynamics.

## 4. Role of mtDNA in Ozone Exposure

As discussed above, mtDNA undergoes significantly weaker quality control compared to the nuclear genome [109]. The release of mtDNA serves as a crucial biomarker for various diseases, including neurodegenerative disorders and certain cardiovascular conditions [75,110]. During O_3_ exposure, mtDNA also functions as an important indicator to assess the severity of tissue damage. A study on middle-aged and elderly individuals at high risk of cardiovascular disease revealed that short-term O_3_ exposure alters mtDNA methylation levels, thereby increasing the likelihood of myocardial ischemia symptoms [111]. Furthermore, a 2022 study simultaneously analyzed the independent and combined effects of environmental O_3_ exposure and residential greening on mitochondrial DNA copy number. It found that long-term O_3_ exposure correlates with altered mitochondrial DNA copy number, while residential greening partially mitigates this association [112]. This raises a critical question: How is released mtDNA associated with O_3_ exposure and inflammatory damage?

### 4.1. Mechanisms of mtDNA Damage in Ozone Exposure

O_3_ is a potent oxidizing agent that can interact with various biomolecules in living organisms, leading to the generation of large amounts of ROS, including superoxide anions and hydroxyl radicals. These substances further damage essential organic macromolecules such as lipids, protein, and DNA [113]. Mitochondria serve as the primary site for ROS production within cells, relying on the cellular intrinsic antioxidant system to maintain REDOX balance. When exposed to O_3_, intracellular antioxidant enzymes and reducing agents like vitamin C and glutathione become severely depleted, disrupting the REDOX equilibrium and triggering oxidative stress [114]. Concurrently, due to the unique localization of mtDNA, it becomes directly susceptible to attack by ROS during oxidative stress, leading to oxidation, modification, and fragmentation [115]. For instance, the hydroxyl radical can induce mtDNA mutations by converting guanine into 8-hydroxy-deoxyguanosine (8-OH-dG), which may mispair with adenine (A) instead of cytosine (C) during mtDNA replication [116]. Furthermore, certain ROS, such as superoxide anions, can directly attack the deoxyribose backbone of mtDNA. Catalyzed by iron or copper ions, hydroxyl radicals generated through Fenton or Haber–Weiss reactions can abstract hydrogen atoms from a carbon atom within deoxyribose, leading to strand breaks and compromising mtDNA integrity [117]. Additionally, the oxidative modification of proteins by O_3_ and ROS impairs mtDNA replication and repair mechanisms. In isolated mitochondria exposed to low concentrations of hydrogen peroxide, the oxidized base excision repair enzyme Ntg1 induces double-strand breaks at the mtDNA replication origin ori5, thereby initiating rolling-circle mtDNA replication [118]. Another study on hydrogen peroxide-treated mouse embryonic fibroblasts revealed significant mtDNA damage, loss, and reduced overall storage capacity [119]. A 2023 longitudinal study indicated that individuals exposed to short-term low levels of O_3_ exhibited decreased mean methylation (%5mC) within the mitochondrial displacement loop (D-loop) region post-exposure, suggesting that O_3_ exposure may impact mitochondrial DNA replication and repair processes [6].

In summary, O_3_ exposure exerts adverse effects on mitochondrial DNA, resulting in mtDNA oxidation, mutations, and fragmentation. Such damage may disrupt normal mtDNA replication and complicate repair processes, thereby increasing mtDNA heterogeneity and triggering abnormal copy number variations.

### 4.2. Source of mtDNA

**Figure 2:**
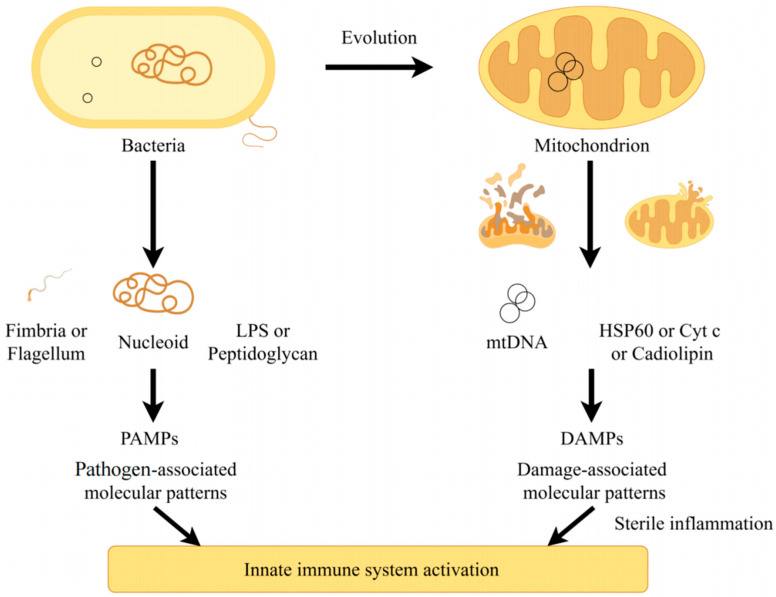
Schematic representation of bacterial and mitochondrial-mediated innate immune responses. On the (**left**), bacterial structures and molecules serve as PAMPs that trigger immune responses; on the (**right**), mtDNA and specific small molecules function as DAMPs, ultimately culminating in aseptic inflammation.

It is widely acknowledged that mitochondria evolved from bacteria engulfed by eukaryotic cells. Therefore, mtDNA shares similarities with bacterial DNA not only in replication mechanisms, structural characteristics, and modification levels but also in immunogenicity. The human body possesses numerous innate immune receptors (i.e., PRRs) capable of recognizing products from invading bacteria or viruses, initiating a cascade of signaling pathways to activate immune cells. These exogenous components derived from bacteria or viruses are termed pathogen-associated molecular patterns (PAMPs). Similarly, mtDNA released from damaged or dysfunctional mitochondria serves as a significant source of damage-associated molecular patterns (DAMPs) [120]. When mtDNA enters the cytoplasm or extracellular matrix, it is recognized by PRRs that activate innate immune responses and inflammation in the absence of infection. Figure 2 illustrates this process. Previous research has demonstrated that sterile inflammation induced by O_3_ exposure is frequently associated with mitochondrial dysfunction [121].

However, mtDNA is typically localized within the nucleoid and its distribution is confined to the mitochondrial matrix. Its release requires either an active regulatory process mediated by a specific mechanism or through a passive accidental pathway.

#### 4.2.1. mtDNA Release Mediated by mPTP

**Figure 3:**
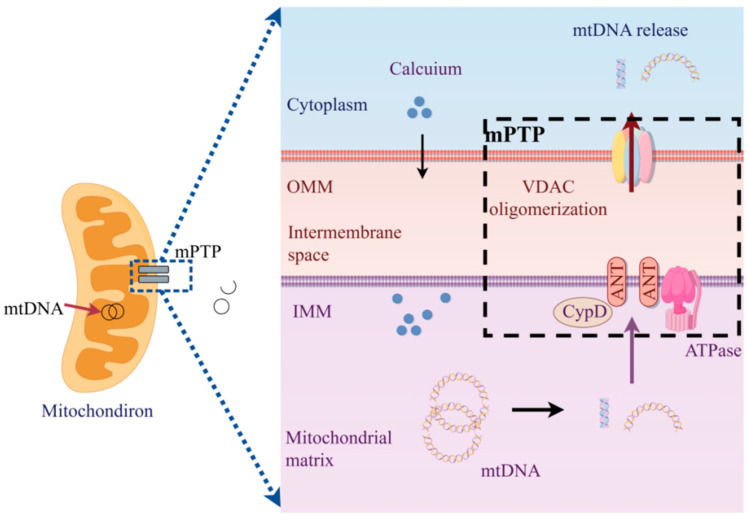
Schematic representation of the structural composition and position of the mitochondrial permeability transition pore (mPTP). This diagram illustrates how the mPTP channel opens following a large influx of calcium ions into the mitochondrial matrix, leading to mitochondrial DNA release.

As shown in Figure 3, the mitochondrial permeability transition pore (mPTP) is a supramolecular structure composed of voltage-dependent anion channels (VDACs), adenine nucleotide translocase (ANT), and cyclophilin D (CyD), collectively mediating mitochondrial permeability [122]. O_3_- and ROS-induced oxidation of lipids and membrane proteins compromises the function and integrity of the mitochondrial membrane, with one of the most evident manifestations being a decrease in mitochondrial membrane potential [123]. This decline triggers the opening of mPTP, serving as a key step in promoting calcium ion influx [124,125]. Furthermore, ROS-induced oxidative modifications may alter the activity of sodium-calcium exchangers (NCLX) and the mitochondrial calcium uniporter (MCU), along with their regulatory proteins, ultimately leading to an overall increase in calcium inflow [126]. Subsequent mitochondrial calcium overload exacerbates mPTP opening, resulting in mitochondrial matrix swelling, membrane potential collapse, oxidative phosphorylation uncoupling, and ultimately triggering apoptosis or cell death [127]. In various cell types, including immune cells, mPTP activation upon stimuli such as lipopolysaccharide, radiation exposure, or oxidative stress facilitates the release of mtDNA fragments into the cytoplasm [128]; these fragments are likely generated through FEN-1 cleavage at approximately 500–650 bp lengths and may contain protein complexes encoding components involved in oxidative phosphorylation such as cytochrome c oxidase subunit 1 (MTCO1), NADH dehydrogenase subunit 3 (MTND3), and partial fragments of cytochrome B [129,130]. This phenomenon elucidates how heightened mtROS levels contribute to reduced metabolic activity. Furthermore, studies have shown that cyclosporin A—a known inhibitor of mPTP—significantly mitigates O_3_-induced conjunctivitis associated with mtDNA release [20].

#### 4.2.2. mtDNA Release Mediated by BAK/BAX

In fact, beyond calcium-dependent opening of mPTP, activation of the Bax/Bak pathway under apoptotic stimuli also enhances the permeability of the outer mitochondrial membrane and facilitates pore formation, ultimately leading to mPTP opening [131]. Bax (Bcl-2-associated X protein) and Bak (Bcl-2 homologous antagonist/killer) are pro-apoptotic members of the Bcl-2 (B-cell lymphoma/leukemia-2) protein family, typically existing in a stable inactive state within cells. Bax is a soluble cytoplasmic protein that, upon activation, is transported to the mitochondrial membrane; Bak, conversely, is an integral protein of the outer mitochondrial membrane [132]. Upon apoptotic signaling, their conformations change, allowing insertion into the mitochondrial outer membrane through multiple α-helical domains to form oligomers and polymer complexes, thereby creating large pores in the membrane [133,134]. With sustained stimulation, these macropores deepen and extend into the inner mitochondrial membrane; mtDNA nuclear regions can be released into the cytoplasm through the formed Bak/Bax pores [135]. However, recent studies have demonstrated that mtDNA release occurs even in cells undergoing apoptosis despite inhibition of mPTP opening by cyclosporine A. This not only indicates that mtDNA nucleoids can be released through Bak/Bax macropores but also suggests that such macropore formation does not necessarily depend on mPTP opening [136]. In studies of lung injury induced by acute O_3_ exposure, excessive ROS was observed to facilitate BAX/BAK upregulation via the mitochondrial pathway, consequently leading to apoptosis. This suggests that released mitochondrial DNA (mtDNA) may play a potential role [137,138]. Furthermore, during Bak/Bax-mediated apoptosis, when cell membranes are compromised by apoptosis-associated degradation processes, mtDNA may leak into circulation and, under sustained stimulation, convert into circulating cell-free mtDNA (ccf-mtDNA) [139,140].

#### 4.2.3. mtDNA Release Mediated by Abnormal Mitochondrial Dynamics

Mitochondrial dynamics encompass both fission and fusion processes, which are essential for maintaining the dynamic equilibrium in mitochondrial quantity and functionality. External stimuli, including O_3_ exposure, can disrupt this delicate balance [141,142]. One study involving O_3_ exposure revealed that acutely exposed mice exhibited impaired mitochondrial fusion capacity alongside airway inflammation and hyperresponsiveness. Similarly, acutely O_3_-exposed BEAS-2B cells demonstrated not only inflammatory responses but also mitochondrial structural and functional abnormalities characterized by reduced fusion capacity [143]. Under oxidative stress from ROS, mtDNA undergoes oxidation, resulting in mutations or fragmentation. Oxidative modifications or calcium influx may abnormally activate replication-associated enzymes, ultimately triggering mtDNA replication stress during abnormal mitochondrial fission [144]. Concurrently, elevated levels of mitochondrial ROS activate ATAD3B as an autophagy-initiating receptor, rather than facilitating mtDNA repair [145]. The release of mtDNA occurs during abnormal processes of both mitochondrial fission and mitophagy [146].

#### 4.2.4. mtDNA Release Mediated by Mitochondrial-Derived Vesicles (MDVs)

Mitochondrial-Derived Vesicles (MDVs) constitute a specialized class of vesicles originating from mitochondria. Their formation mechanism is complex, and they play a crucial role in mitochondrial quality control and signal transduction [147]. MDVs encapsulate damaged mitochondrial components, facilitating their transport to lysosomes or proteasomes for degradation, thereby preserving the quality and functional integrity of mitochondria [148]. Through continuous generation and release, MDVs also critically regulate mitochondrial size and morphology, enabling adaptation to diverse cellular states such as stress responses or during cell growth and division [149]. Current studies confirm that fragments of mtDNA can be released into the cytoplasm through MDVs, a process independent of mitochondrial autophagy or fission processes [150,151]. In response to mitochondrial stress, nucleoids containing mtDNA are selectively transferred to these vesicles, a process potentially involving cardiolipin oxidation. Under conditions stimulated by ROS or other factors, cardiolipin oxidation generates phosphatidic acid, altering mitochondrial membrane curvature and inducing outward mitochondria deformation [152,153]. As a consequence of this morphological change, mitochondria-derived vesicles ultimately mature with the assistance of PINK1 and Parkin proteins [154]. These vesicles subsequently migrate towards the endoplasmic reticulum to activate the corresponding signal pathways, after which they are directed toward lysosomes or released extracellularly [155].

## 5. Released mtDNA Induces Inflammatory Response

Currently, it is widely accepted that mitochondria originated from an alpha-proteobacterium engulfed by eukaryotic host cells [156]. Over long-term evolution, host cells and mitochondria mutually adapted, establishing intricate signaling pathways [157]. However, mitochondria retain certain ancestral bacterial characteristics, such as a double-layer membrane structure, unique cardiolipin in the mitochondrial membrane, and distinct replication and transcription modes from the nuclear genome. These factors contribute to mitochondrial heterogeneity and can trigger immunogenicity of mitochondrial components under specific conditions [158]. Similarly, due to its bacterial derivation and the presence of hypomethylated CpG sequences, mtDNA functions as a damage-associated molecular pattern (DAMP) discharged by mitochondria, thereby serving as a robust trigger for innate immune response [159]. mtDNA has been shown to induce inflammation responses by interacting with pattern recognition receptors (PRRs), including TLR9, NLRP3, and the cGAS-STING pathway [129,160,161].

**Figure 4:**
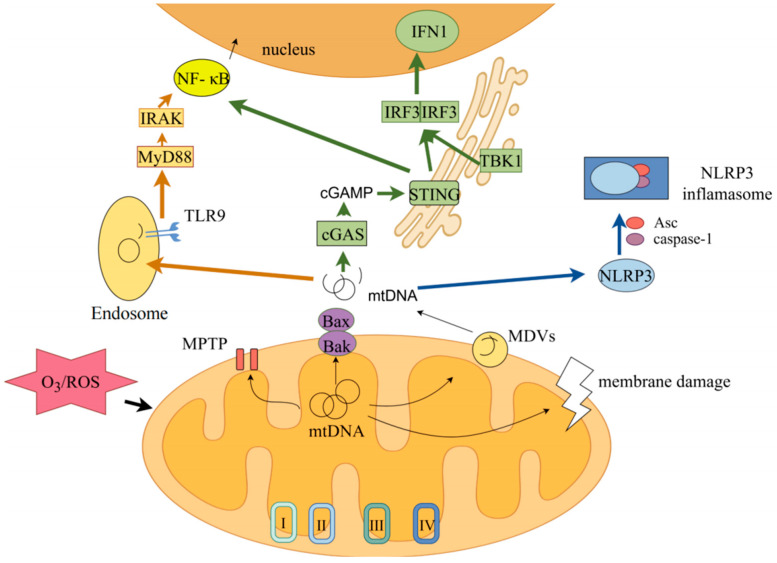
Schematic representation of mtDNA release and activation of downstream pathways following ozone exposure. Ozone exposure mediates mitochondrial release of mtDNA. mtDNA-cGAS-STING pathway (labeled in green). mtDNA-TLR9/4-MyD88-NF-κB (labeled in orange). mtDNA-NLRP3-ASC-Caspase1 pathway (labeled in blue). Produced with Figdraw.

The mechanism through which ozone exposure induces mtDNA release and subsequently activates downstream inflammatory pathways is illustrated in Figure 4.

### 5.1. cGAS-STING Pathway

The investigation of the cGAS-STING signaling pathway is an ongoing process of in-depth exploration. First reported in 2013, the mechanism underlying its interaction with cytoplasmic DNA and subsequent activation has been elucidated [162]. The cGAS-STING pathway primarily consists of cyclic GMP-AMP synthase (cGAS) and Stimulator of Interferon Genes (STING). As a cytoplasmic protein, cGAS possesses the characteristic structure of the cGAS/DNCV-like nucleotide transferase (CD-NTases) family. Its overall structure comprises two distinct domains: the nucleotide transferase domain and the DNA-binding domain [163]. The nucleotide transferase domain possesses a highly conserved three-dimensional spatial structure, forming a cage-like or pocket-like structure whose size, shape, and chemical properties complement the structure of the substrate molecule, ensuring precise and efficient entry and binding at the active site. It typically adopts a conformation capable of binding to the surface of the DNA double helix, allowing it to better accommodate the major or minor grooves of DNA for specific binding [164]. Through these two distinct DNA recognition sites, cGAS can effectively identify and bind to dsDNA, thereby initiating its catalytic activity to produce cyclic guanosine monophosphate–adenosine monophosphate synthase (cGAMP) [165]. Cyclic guanosine monophosphate (cGAMP), as an endogenous signaling molecule, activates STING receptors situated in the endoplasmic reticulum. This leads to the phosphorylation of tank binding kinase 1 (TBK1) and interferon regulatory factor 3 (IRF3), ultimately mediating the transcription of interferon-stimulated genes (ISGs) and type I interferon (IFN-1) [166]. Upregulation of IFN-1 promotes the upregulation of tumor necrosis factor α (TNF-α), thereby enhancing the inflammatory response [167]. Concurrently, STING also has the capacity to stimulate the NF-κB signaling cascade, facilitating the synthesis of proinflammatory cytokines [168].

The cGAS-STING signaling pathway has been demonstrated to participate in diverse physiological and pathological processes, emerging as a critical regulatory target in tumorigenesis, autoimmune diseases, infectious diseases, and neurodegenerative disorders. Currently, technical challenges associated with O_3_ exposure hinder comprehensive long-term research on its role in mediating inflammation responses. Nonetheless, existing evidence suggests that direct O_3_ exposure can induce mtDNA release, thereby activating the cGAS-STING signaling pathway and contributing to the pathogenesis of conjunctivitis [20]. In studies examining the intestines of Wistar rats exposed to O_3_, it was also observed that O_3_ exposure facilitated the translocation of NF-κB into the nucleus and modulated its activity, resulting in altered intestinal permeability and fostering chronic inflammation. This further indicates the importance of cGAS-STING to activate NF-κB [169].

### 5.2. TLR9/4-MyD88-NF-κB Pathway

The Toll-like receptor (TLR) family constitutes a class of crucial pattern recognition receptors capable of identifying various bacterial signatures to initiate an immune response. TLR9 was the first receptor reported to recognize unmethylated CpG-DNA [170]. Regarding TLR4, although no direct evidence supports its ability to recognize free fragments of mtDNA, studies have indicated that mtDNA damage frequently accompanies TLR4 activation [171,172]. Both TLR9 and TLR4 are type 1 transmembrane proteins whose structures can be divided into three distinct domains: extracellular domain, transmembrane segment, and intracellular domain. The N-terminal region of each protein’s extracellular domain contains leucine-rich repeat (LRR) sequences, which specifically recognize and bind PAMPs or DAMPs. The intracellular domain features a Toll/IL-1 receptor (TIR) domain common among members of the TLR family, facilitating interactions with downstream adaptor proteins such as myeloid differentiation factor 88 (MyD88), thereby initiating subsequent signaling cascades [173].

TLR4 is predominantly expressed on the cell membrane of immune cells and certain endothelial cells, including macrophages, dendritic cells, and respiratory epithelial cells [174,175,176]. In contrast, TLR9 is primarily localized within the endoplasmic reticulum, endosomes, lysosomes, and other organelles of immune cells, as well as plasmacytoid dendritic cells (pDCs) and B lymphocytes [177,178,179]. At rest, TLR9 is typically localized in the endoplasmic reticulum; upon recognition of unmethylated CpG sequences via endocytosis, TLR9 is transferred to the endosome, where MyD88 is recruited and IRAK is activated, leading to NF-κB dephosphorylation [180]. The released NF-κB then translocates into the nucleus to exert its biological functions, thereby upregulating pro-IL-1β and pro-IL-18 expression [181,182]. Furthermore, the activation of NF-κB facilitates the interaction with interferon regulatory factors (e.g., IRF3 and IRF7), collectively driving type I interferon response elements, enhancing cellular immunity, and upregulating TNF-α expression [183]. Despite differing distribution patterns, TLR4 similarly recruits MyD88 to regulate downstream signaling pathways [184,185]. Previous studies indicate that TLR4 synergizes with TLR9 to amplify inflammatory responses [186].

### 5.3. NLRP3-ASC-Caspase1 Pathway

Another inflammatory response closely associated with mtDNA release is the activation of nucleotide-binding oligomerization domain (NOD)-like receptors. The NOD-like receptor family pyrin domain containing 3 (NLRP3), as a pattern recognition receptor, recognizes various pathogen-associated molecular patterns (PAMPs) and damage-associated molecular patterns (DAMPs). In its quiescent state, NLRP3 remains inactive; however, upon stimulation, it synergistically activates with NEK7, thereby facilitating NLRP3 inflammasome assembly [187]. The NLRP3 inflammasome is a cytoplasmic multiprotein complex composed of the NLRP3 sensor protein, apoptosis-associated speck-like protein (ASC), and pro-caspase-1. One end of the ASC protein binds to the NLRP3 PYD domain via its homotypic interaction domain (PYD), while the other end binds to the caspase-1 precursor through the caspase recruitment domain (CARD), thereby establishing a connection between NLRP3 and pro-caspase-1 [188]. The recruited inactive pro-caspase-1 undergoes self-cleavage to generate active caspase-1, which subsequently initiates the maturation and secretion of pro-inflammatory cytokines IL-1β and IL-18 [189].

The NLRP3 inflammasome is extensively distributed across various immune and non-hematopoietic cell types, including macrophages, dendritic cells, and epithelial cells [190,191,192]. When oxidized mitochondrial DNA (Ox-mtDNA) is present in the cytoplasm, it is activated and assembled into a fully functional NLRP3 inflammasome [193]. Furthermore, unlike the aforementioned pathways, NLRP3 exhibits a stronger propensity to recognize oxidized mtDNA, indicating the pivotal role of ROS in the activation of the NLRP3 inflammasome [194]. The potential involvement of mitochondria in NLRP3 inflammasome activation was first proposed in 2010. This study revealed that inhibiting mitochondrial autophagy results in an accumulation of damaged mitochondria, which in turn generates high levels of ROS, thereby activating the inflammasome [195]. Notably, beyond serving as a downstream signal for mtDNA release, NLRP3 also exerts upstream regulation by facilitating VDAC oligomerization and opening calcium-dependent mPTP channels, thereby releasing fragmented Ox-mtDNA and increasing ROS production [196,197]. These leaked oxidized mtDNA further promote NLRP3 activation, intensifying the inflammatory response. In numerous O_3_ exposure experiments, NLRP3 inflammasome activation has frequently been identified as a significant upstream inflammatory factor, typically associated with compromised mitochondrial function and ROS accumulation [198]. Research has also found that TLR4 and NLRP3 activation correlates with O_3_ exposure [199]. Unfortunately, a direct experimental link between oxidized mtDNA and NLRP3 remains to be established.

In summary, the released mtDNA can be recognized by multiple pattern recognition receptors (PRRs), playing a crucial role in activating inflammatory responses through innate immune pathways, including mtDNA-cGAS-STING, mtDNA-TLR9/4-MyD88-NF-κB, and mtDNA-NLRP3-ASC-Caspase1.

### 5.4. The Regulatory Relationship Among Ozone Exposure, mtDNA, and Inflammatory Response

Ozone exposure induces oxidative stress, damaging mitochondrial membranes and leading to the release of mitochondrial DNA (mtDNA) into the cytoplasm and extracellular space [6]. Once released, mtDNA acts as a damage-associated molecular pattern (DAMP) that activates multiple innate immune signaling pathways [42]. In the cytoplasm, mtDNA binds to cyclic GMP-AMP synthase (cGAS), activating the stimulator of interferon genes (STING) pathway to produce type I interferons via the TBK1-IRF3 signaling pathway [200]. Concurrently, extracellular mtDNA activates Toll-like receptor 9 (TLR9) or TLR4 through unmethylated CpG motifs, triggering MyD88-dependent NF-κB activation and pro-inflammatory cytokine transcription [201]. Furthermore, oxidized mtDNA (ox-mtDNA) fragments directly bind the NLRP3 inflammasome, recruiting ASC and activating caspase-1 to cleave pro-IL-1β and pro-IL-18, ultimately inducing pyroptosis [131,202]. These parallel signaling cascades establish a robust inflammatory response that mediates ozone-induced tissue damage in the lungs and systemic organs [6,131].

## 6. Conclusions

Currently, research on O_3_ exposure remains relatively limited; however, due to inherent limitations in O_3_ exposure methodologies, the establishment of O_3_ toxicology and in vivo exposure studies requires further development and refinement. Epidemiological evidence indicates that O_3_ exposure is associated with increased risks of various neurological, respiratory, and cardiovascular diseases, often accompanied by elevated inflammatory markers and mitochondrial dysfunction. Furthermore, existing studies on O_3_ exposure have provided important findings regarding mtDNA copy number and dynamics. These investigations have also explored downstream pathway activation mechanisms involving mtDNA, such as the NLRP3 inflammasome and NF-κB pathways, suggesting a potential role for mtDNA in these processes. Although experimental evidence remains incomplete, emerging indications suggest that O_3_ exposure can induce conjunctivitis by releasing mtDNA as a danger-associated molecular pattern (DAMP), thereby activating the cGAS-STING signaling pathway. By integrating existing O_3_ epidemiological analyses, toxicological data, and a comprehensive ROS mechanism study, we confirm mtDNA as a pivotal molecule linking in vivo O_3_ exposure to inflammation. Its release may function as a DAMP—an innate immune signal that promotes pathways including cGAS-STING, TLR9/4-MyD88-NF-κB, and NLRP3-ASC-Caspase1.

However, it must be acknowledged that the current number of relevant toxicological experiments remains insufficient to determine the specific pathways through which mtDNA downstream activation occurs under O_3_ exposure conditions. Additionally, further experimental validation is required to elucidate how mtDNA is released from mitochondria and its subsequent existence as free-floating molecules within the cytoplasm or as extracellular entities. This would significantly enhance our understanding of the internal mechanisms of O_3_ exposure and contribute to establishing a more precise O_3_ internal exposure–response relationship.

## Data Availability

No new data were created or analyzed in this study. Data sharing is not applicable to this article.
